# 2521. VE707, a Defined Live Biotherapeutic Product for Prevention of Infection by Multidrug-Resistant Gram-Negative Bacteria

**DOI:** 10.1093/ofid/ofad500.2139

**Published:** 2023-11-27

**Authors:** Gregory Medlock, Cintia Felix, Wajd Alsharif, Lou Cornacchione, Matthew Schinn, Andrea Watson, Suzanne Bedard-Shurtleff, Jason Norman, Jeremiah Faith, Ed J Kuijper, Bernat Olle, Silvia Caballero

**Affiliations:** Vedanta Biosciences, Cambridge, Massachusetts; Vedanta Biosciences, Cambridge, Massachusetts; Vedanta Biosciences, Cambridge, Massachusetts; Vedanta Biosciences, Cambridge, Massachusetts; Vedanta Biosciences, Cambridge, Massachusetts; Vedanta Biosciences, Cambridge, Massachusetts; Vedanta Biosciences, Cambridge, Massachusetts; Vedanta Biosciences, Cambridge, Massachusetts; Icahn School of Medicine at Mount Sinai, New York, New York; Leiden University Medical Center and RIVM, Leiden, Zuid-Holland, Netherlands; Vedanta Biosciences, Cambridge, Massachusetts; Vedanta Biosciences, Cambridge, Massachusetts

## Abstract

**Background:**

Infections with multidrug-resistant organisms (MDRO) are increasing at an alarming rate in hospitals worldwide. MDRO infections are often preceded by asymptomatic intestinal colonization and growth by the MDRO. Treatments such as non-absorbable antibiotics and fecal microbiota transplantation (FMT), which decrease the abundance of MDRO in the intestines, have shown efficacy at preventing MDRO infection. Despite the success of FMT at reducing intestinal MDRO abundance without leading to resistance, FMT composition and efficacy is variable and its safety profile questionable. This highlights the need for a defined microbiome-based product with robust efficacy and standardized manufacturing.

**Methods:**

VE707 is a defined live biotherapeutic product (LBP) consisting of a consortium of bacterial strains that reduce intestinal carriage of carbapenem-resistant and extended-spectrum beta-lactamase-producing *Klebsiella pneumoniae* (Kpn) and *Escherichia coli* (Eco). Using a top-down approach, we characterized fecal material from healthy individuals for their ability to suppress Kpn and Eco *ex vivo* and *in vivo*, then identified donor material enriched for activity against both pathogens. Next, we used *in vitro*, *in vivo*, and bioinformatic approaches to design defined LBPs using the bacterial strains from this donor based on their anti-MDRO activity. We evaluated the ability of these LBPs to reduce Kpn and Eco abundance in a mouse co-colonization model.

**Results:**

Of 94 LBPs designed and evaluated which each consisted of between 7 and 50 strains, VE707 showed the greatest activity, as demonstrated by a > 3-log10 reduction in Kpn and Eco in stool (Figure 1; *p* = 0.0022). Furthermore, VE707 was active *in vitro* against a panel of 40 MDR Kpn and Eco clinical isolates ( > 2-log10 reduction in Kpn and Eco growth, *p* < 0.05). We have developed a co-culture process to enable commercially feasible manufacturing of VE707 and found that VE707 produced via co-culture has *in vivo* anti-MDRO activity equivalent to VE707 produced via monoculture (Figure 1; > 3-log10 reduction in Kpn and Eco in stool, *p* = 0.0022).

**Figure 1.**
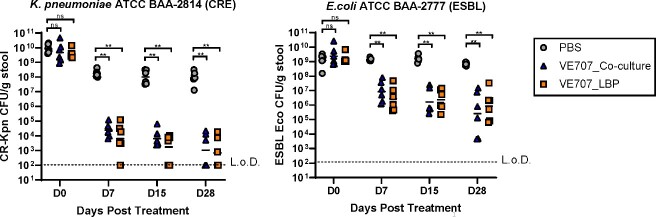
Treatment with VE707, produced via monoculture or co-culture, reduces stool Kpn and Eco titer by > 3-log10 in a mouse model of co-colonization.

Left: VE707 reduces the abundance of Klebsiella pneumoniae ATCC BAA-2814, a model carbapenem-resistant Enterobacteriaceae (CRE) species. Right: VE707 reduces the abundance of Escherichia coli ATCC BAA-2777, a model extended-spectrum beta-lactamase (ESBL)-producing Enterobacteriaceae species. Pathogen decolonization was determined using a murine co-colonization model with simultaneous Kpn and Eco gavage. VE707 produced via monoculture fermentation (VE707_LBP) and via co-culture fermentation (VE707_Co-culture) were evaluated. Black horizontal bars show geometric mean. **: p = 0.0022, ns = not significant, tested via Mann-Whitney U-test. L. o. D., limit of detection. VE707_LBP = VE707 produced via monoculture of each strain in the consortia. VE707_Co-culture = VE707 produced via a co-culture fermentation process. PBS = treatment with phosphate buffered saline.

**Conclusion:**

Our results show that VE707, a defined bacterial consortium with broad anti-MDRO activity, is successful at decolonizing Kpn and Eco and can be manufactured efficiently with a co-culture process.

**Disclosures:**

**Gregory Medlock, PhD**, Vedanta Biosciences: Ownership Interest **Cintia Felix, MS**, Vedanta Biosciences: Ownership Interest **Lou Cornacchione, PhD**, Vedanta Biosciences: Ownership Interest **Matthew Schinn, PhD**, Vedanta Biosciences: Ownership Interest **Andrea Watson, PhD**, Vedanta Biosciences: Advisor/Consultant|Vedanta Biosciences: Honoraria **Jason Norman, PhD**, Vedanta Biosciences: Patent Inventor|Vedanta Biosciences: Employee, Stock Options **Jeremiah Faith, PhD**, Vedanta Biosciences: Advisor/Consultant **Ed J. Kuijper, Prof. Dr.**, Vedanta Biosciences: Advisor/Consultant **Bernat Olle, PhD**, Vedanta Biosciences, Inc.: Board Member|Vedanta Biosciences, Inc.: Employee, receive salary and equity compensation|Vedanta Biosciences, Inc.: Ownership Interest **Silvia Caballero, PhD**, Vedanta Biosciences: Advisor/Consultant|Vedanta Biosciences: Board Member|Vedanta Biosciences: Ownership Interest

